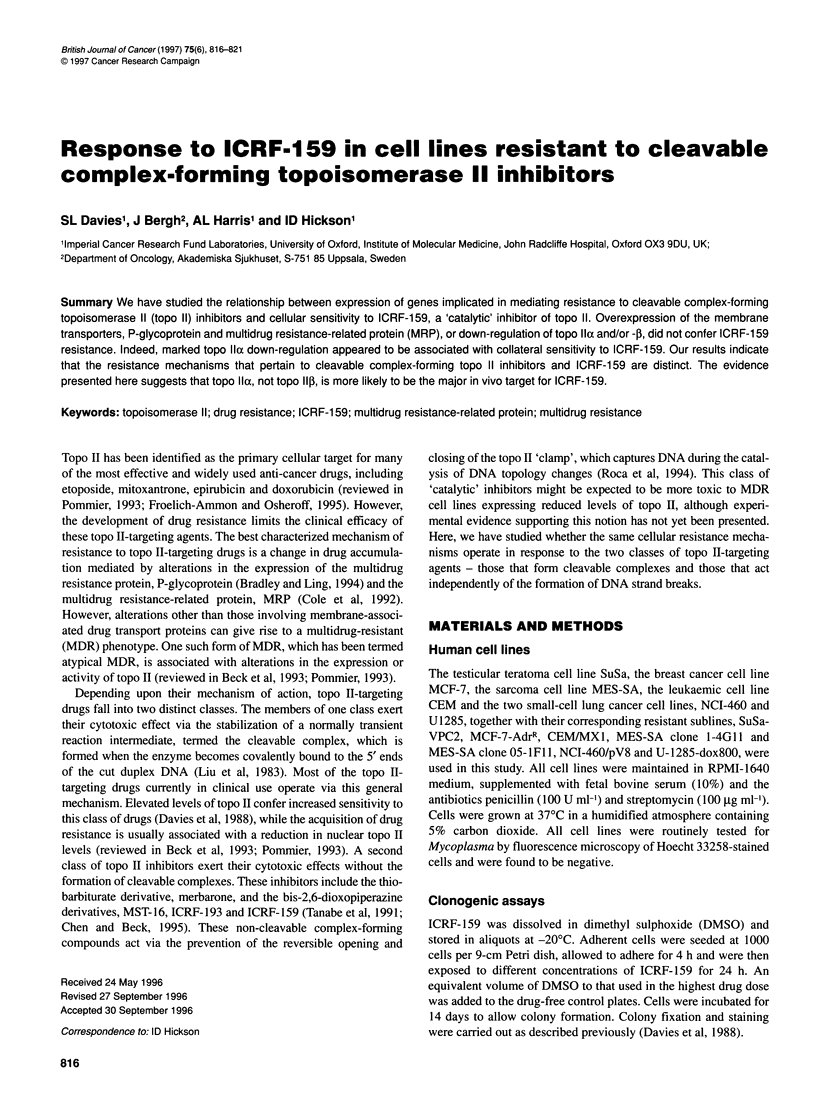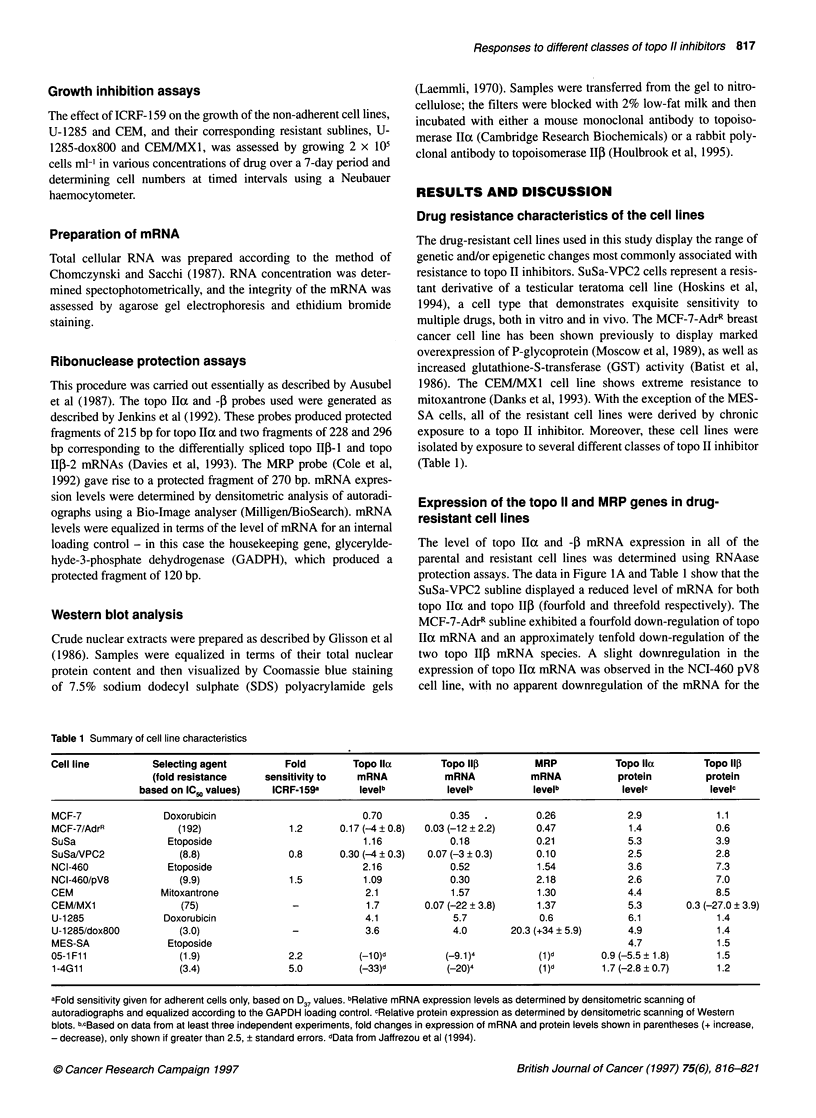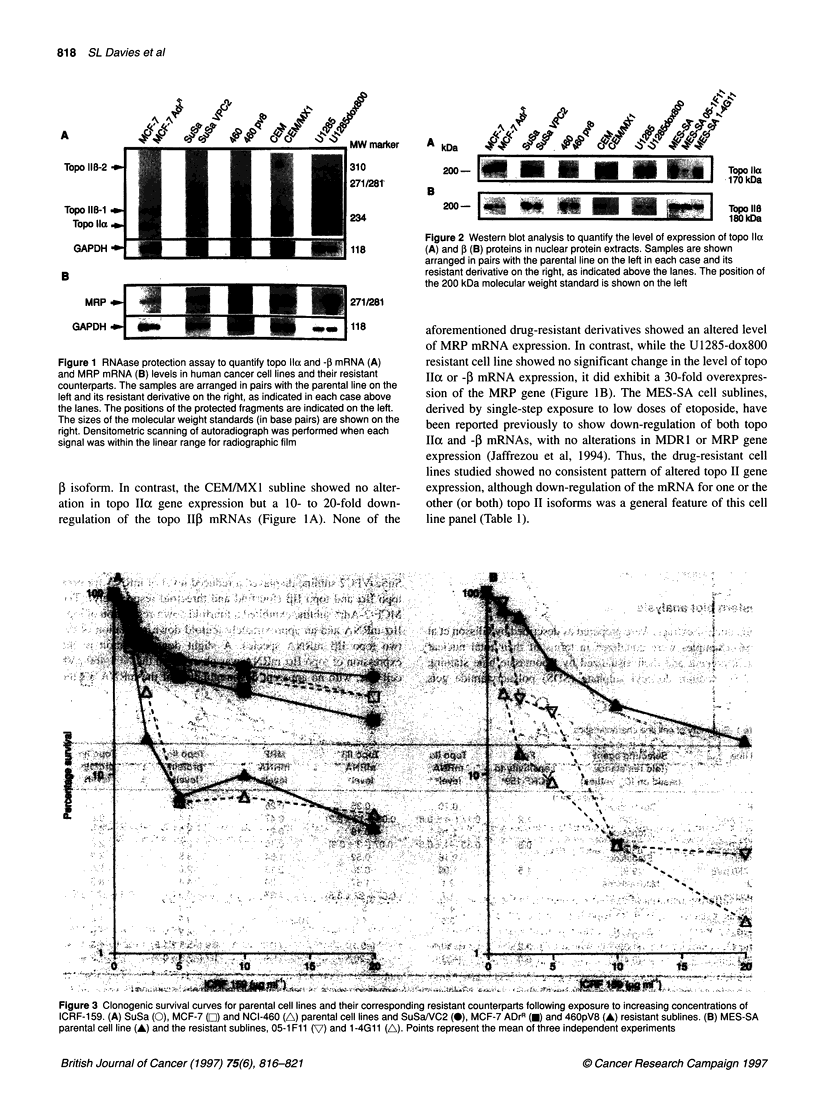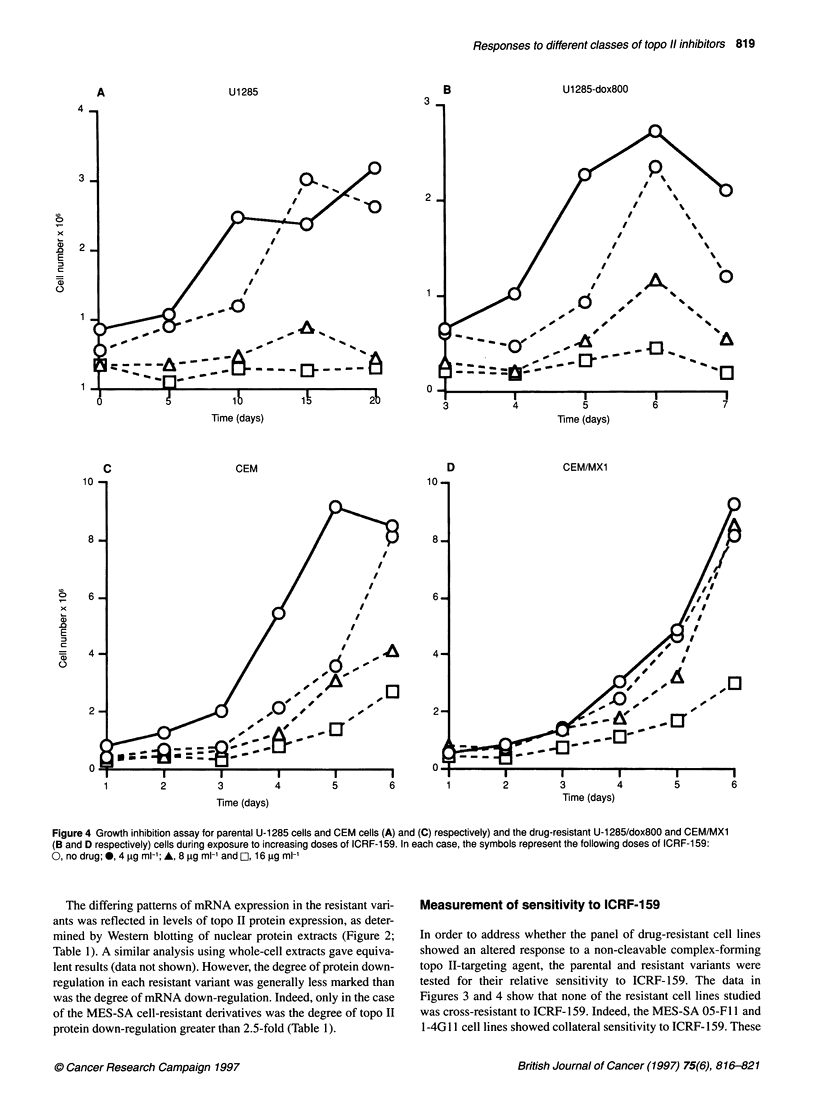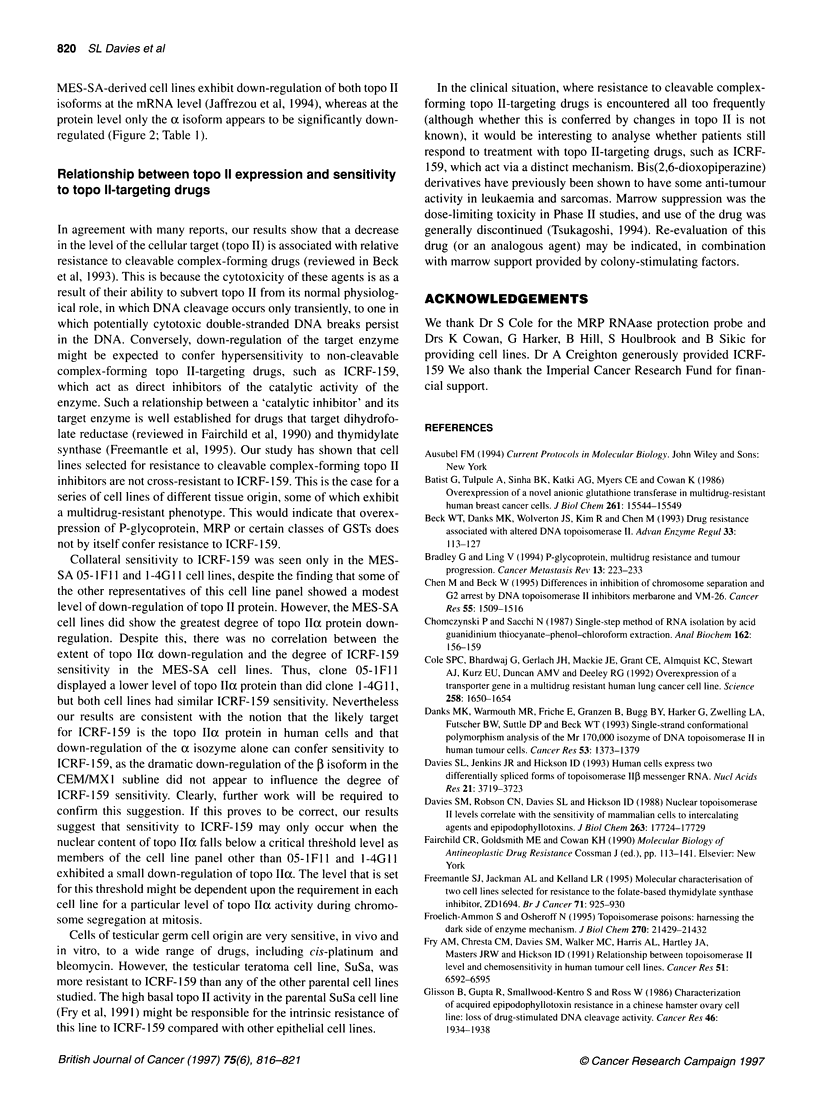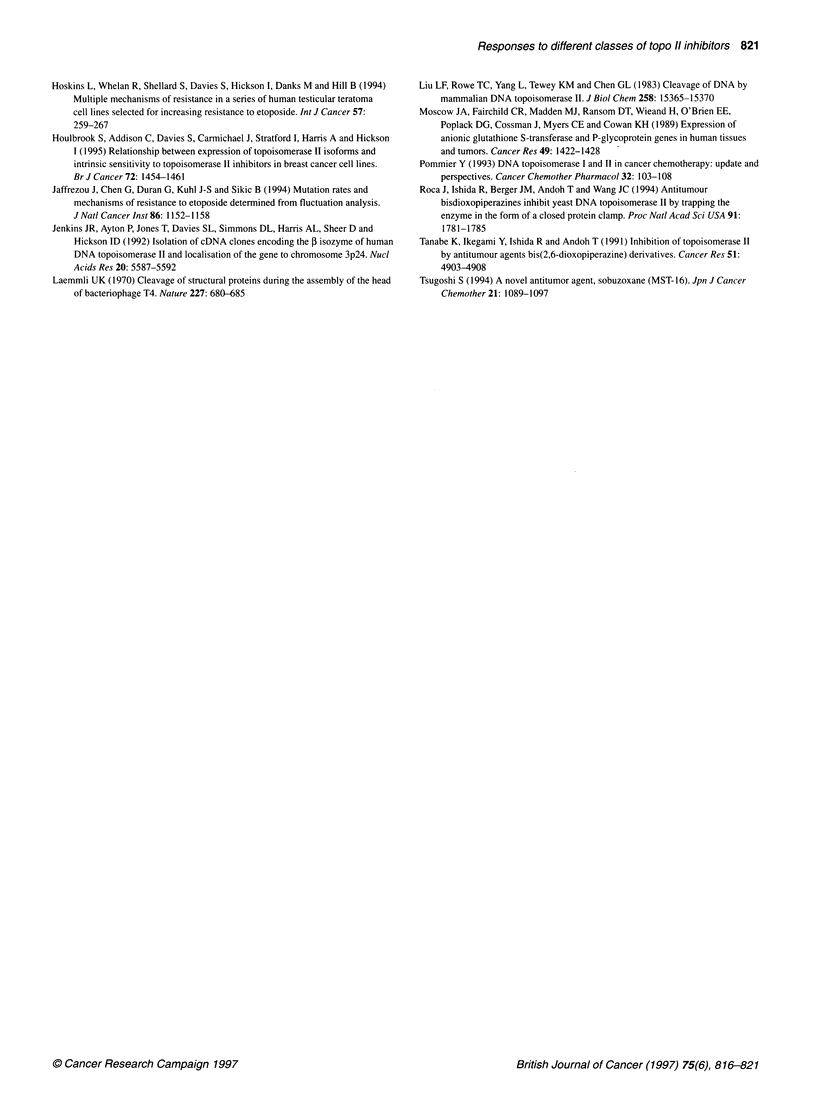# Response to ICRF-159 in cell lines resistant to cleavable complex-forming topoisomerase II inhibitors.

**DOI:** 10.1038/bjc.1997.146

**Published:** 1997

**Authors:** S. L. Davies, J. Bergh, A. L. Harris, I. D. Hickson

**Affiliations:** Imperial Cancer Research Fund Laboratories, University of Oxford, UK.

## Abstract

**Images:**


British Journal of Cancer (1997) 75(6), 816–821
© 1997 Cancer Research Campaign

### Response to ICRF-159 in cell lines resistant to cleavable complex-forming topoisomerase II inhibitors

**SL Davies[1], J Bergh[2], AL Harris[1] and ID Hickson[1]**

[1]Imperial Cancer Research Fund Laboratories, University of Oxford, Institute of Molecular Medicine, John Radcliffe Hospital, Oxford OX3 9DU, UK;
[2]Department of Oncology, Akademiska Sjukhuset, S-751 85 Uppsala, Sweden

**Summary** We have studied the relationship between expression of genes implicated in mediating resistance to cleavable complex-forming topoisomerase II (topo II) inhibitors and cellular sensitivity to ICRF-159, a 'catalytic' inhibitor of topo II. Overexpression of the membrane transporters, P-glycoprotein and multidrug resistance-related protein (MRP), or down-regulation of topo IIα and/or -β, did not confer ICRF-159 resistance. Indeed, marked topo IIα down-regulation appeared to be associated with collateral sensitivity to ICRF-159. Our results indicate that the resistance mechanisms that pertain to cleavable complex-forming topo II inhibitors and ICRF-159 are distinct. The evidence presented here suggests that topo IIα, not topo IIβ, is more likely to be the major in vivo target for ICRF-159.

**Keywords:** topoisomerase II; drug resistance; ICRF-159; multidrug resistance-related protein; multidrug resistance

Topo II has been identified as the primary cellular target for many of the most effective and widely used anti-cancer drugs, including etoposide, mitoxantrone, epirubicin and doxorubicin (reviewed in Pommier, 1993; Froelich-Ammon and Osheroff, 1995). However, the development of drug resistance limits the clinical efficacy of these topo II-targeting agents. The best characterized mechanism of resistance to topo II-targeting drugs is a change in drug accumulation mediated by alterations in the expression of the multidrug resistance protein, P-glycoprotein (Bradley and Ling, 1994) and the multidrug resistance-related protein, MRP (Cole et al, 1992). However, alterations other than those involving membrane-associated drug transport proteins can give rise to a multidrug-resistant (MDR) phenotype. One such form of MDR, which has been termed atypical MDR, is associated with alterations in the expression or activity of topo II (reviewed in Beck et al, 1993; Pommier, 1993).

Depending upon their mechanism of action, topo II-targeting drugs fall into two distinct classes. The members of one class exert their cytotoxic effect via the stabilization of a normally transient reaction intermediate, termed the cleavable complex, which is formed when the enzyme becomes covalently bound to the 5′ ends of the cut duplex DNA (Liu et al, 1983). Most of the topo II-targeting drugs currently in clinical use operate via this general mechanism. Elevated levels of topo II confer increased sensitivity to this class of drugs (Davies et al, 1988), while the acquisition of drug resistance is usually associated with a reduction in nuclear topo II levels (reviewed in Beck et al, 1993; Pommier, 1993). A second class of topo II inhibitors exert their cytotoxic effects without the formation of cleavable complexes. These inhibitors include the thiobarbiturate derivative, merbarone, and the bis-2,6-dioxopiperazine derivatives, MST-16, ICRF-193 and ICRF-159 (Tanabe et al, 1991; Chen and Beck, 1995). These non-cleavable complex-forming compounds act via the prevention of the reversible opening and closing of the topo II 'clamp', which captures DNA during the catalysis of DNA topology changes (Roca et al, 1994). This class of 'catalytic' inhibitors might be expected to be more toxic to MDR cell lines expressing reduced levels of topo II, although experimental evidence supporting this notion has not yet been presented. Here, we have studied whether the same cellular resistance mechanisms operate in response to the two classes of topo II-targeting agents – those that form cleavable complexes and those that act independently of the formation of DNA strand breaks.

Received 24 May 1996
Revised 27 September 1996
Accepted 30 September 1996

*Correspondence to:* ID Hickson

### MATERIALS AND METHODS

### Human cell lines

The testicular teratoma cell line SuSa, the breast cancer cell line MCF-7, the sarcoma cell line MES-SA, the leukaemic cell line CEM and the two small-cell lung cancer cell lines, NCI-460 and U1285, together with their corresponding resistant sublines, SuSa-VPC2, MCF-7-Adr$^R$, CEM/MX1, MES-SA clone 1-4G11 and MES-SA clone 05-1F11, NCI-460/pV8 and U-1285-dox800, were used in this study. All cell lines were maintained in RPMI-1640 medium, supplemented with fetal bovine serum (10%) and the antibiotics penicillin (100 U $ml^{-1}$) and streptomycin (100 μg $ml^{-1}$). Cells were grown at 37°C in a humidified atmosphere containing 5% carbon dioxide. All cell lines were routinely tested for *Mycoplasma* by fluorescence microscopy of Hoecht 33258-stained cells and were found to be negative.

### Clonogenic assays

ICRF-159 was dissolved in dimethyl sulphoxide (DMSO) and stored in aliquots at –20°C. Adherent cells were seeded at 1000 cells per 9-cm Petri dish, allowed to adhere for 4 h and were then exposed to different concentrations of ICRF-159 for 24 h. An equivalent volume of DMSO to that used in the highest drug dose was added to the drug-free control plates. Cells were incubated for 14 days to allow colony formation. Colony fixation and staining were carried out as described previously (Davies et al, 1988).

### Growth inhibition assays

The effect of ICRF-159 on the growth of the non-adherent cell lines, U-1285 and CEM, and their corresponding resistant sublines, U-1285-dox800 and CEM/MX1, was assessed by growing $2 \times 10^5$ cells $ml^{-1}$ in various concentrations of drug over a 7-day period and determining cell numbers at timed intervals using a Neubauer haemocytometer.

### Preparation of mRNA

Total cellular RNA was prepared according to the method of Chomczynski and Sacchi (1987). RNA concentration was determined spectophotometrically, and the integrity of the mRNA was assessed by agarose gel electrophoresis and ethidium bromide staining.

### Ribonuclease protection assays

This procedure was carried out essentially as described by Ausubel et al (1987). The topo IIα and -β probes used were generated as described by Jenkins et al (1992). These probes produced protected fragments of 215 bp for topo IIα and two fragments of 228 and 296 bp corresponding to the differentially spliced topo IIβ-1 and topo IIβ-2 mRNAs (Davies et al, 1993). The MRP probe (Cole et al, 1992) gave rise to a protected fragment of 270 bp. mRNA expression levels were determined by densitometric analysis of autoradiographs using a Bio-Image analyser (Milligen/BioSearch). mRNA levels were equalized in terms of the level of mRNA for an internal loading control – in this case the housekeeping gene, glycerylde-hyde-3-phosphate dehydrogenase (GADPH), which produced a protected fragment of 120 bp.

### Western blot analysis

Crude nuclear extracts were prepared as described by Glisson et al (1986). Samples were equalized in terms of their total nuclear protein content and then visualized by Coomassie blue staining of 7.5% sodium dodecyl sulphate (SDS) polyacrylamide gels (Laemmli, 1970). Samples were transferred from the gel to nitrocellulose; the filters were blocked with 2% low-fat milk and then incubated with either a mouse monoclonal antibody to topoisomerase IIα (Cambridge Research Biochemicals) or a rabbit polyclonal antibody to topoisomerase IIβ (Houlbrook et al, 1995).

### RESULTS AND DISCUSSION

### Drug resistance characteristics of the cell lines

The drug-resistant cell lines used in this study display the range of genetic and/or epigenetic changes most commonly associated with resistance to topo II inhibitors. SuSa-VPC2 cells represent a resistant derivative of a testicular teratoma cell line (Hoskins et al, 1994), a cell type that demonstrates exquisite sensitivity to multiple drugs, both in vitro and in vivo. The MCF-7-Adr$^R$ breast cancer cell line has been shown previously to display marked overexpression of P-glycoprotein (Moscow et al, 1989), as well as increased glutathione-S-transferase (GST) activity (Batist et al, 1986). The CEM/MX1 cell line shows extreme resistance to mitoxantrone (Danks et al, 1993). With the exception of the MES-SA cells, all of the resistant cell lines were derived by chronic exposure to a topo II inhibitor. Moreover, these cell lines were isolated by exposure to several different classes of topo II inhibitor (Table 1).

### Expression of the topo II and MRP genes in drug-resistant cell lines

The level of topo IIα and -β mRNA expression in all of the parental and resistant cell lines was determined using RNAase protection assays. The data in Figure 1A and Table 1 show that the SuSa-VPC2 subline displayed a reduced level of mRNA for both topo IIα and topo IIβ (fourfold and threefold respectively). The MCF-7-Adr$^R$ subline exhibited a fourfold down-regulation of topo IIα mRNA and an approximately tenfold down-regulation of the two topo IIβ mRNA species. A slight downregulation in the expression of topo IIα mRNA was observed in the NCI-460 pV8 cell line, with no apparent downregulation of the mRNA for the

**Table 1** Summary of cell line characteristics

| Cell line | Selecting agent (fold resistance based on $IC_{50}$ values) | Fold sensitivity to ICRF-159[a] | Topo IIα mRNA level[b] | Topo IIβ mRNA level[b] | MRP mRNA level[b] | Topo IIα protein level[c] | Topo IIβ protein level[c] |
|---|---|---|---|---|---|---|---|
| MCF-7 | Doxorubicin | | 0.70 | 0.35 | 0.26 | 2.9 | 1.1 |
| MCF-7/Adr$^R$ | (192) | 1.2 | 0.17 (–4 ± 0.8) | 0.03 (–12 ± 2.2) | 0.47 | 1.4 | 0.6 |
| SuSa | Etoposide | | 1.16 | 0.18 | 0.21 | 5.3 | 3.9 |
| SuSa/VPC2 | (8.8) | 0.8 | 0.30 (–4 ± 0.3) | 0.07 (–3 ± 0.3) | 0.10 | 2.5 | 2.8 |
| NCI-460 | Etoposide | | 2.16 | 0.52 | 1.54 | 3.6 | 7.3 |
| NCI-460/pV8 | (9.9) | 1.5 | 1.09 | 0.30 | 2.18 | 2.6 | 7.0 |
| CEM | Mitoxantrone | | 2.1 | 1.57 | 1.30 | 4.4 | 8.5 |
| CEM/MX1 | (75) | – | 1.7 | 0.07 (–22 ± 3.8) | 1.37 | 5.3 | 0.3 (–27.0 ± 3.9) |
| U-1285 | Doxorubicin | | 4.1 | 5.7 | 0.6 | 6.1 | 1.4 |
| U-1285/dox800 | (3.0) | – | 3.6 | 4.0 | 20.3 (+34 ± 5.9) | 4.9 | 1.4 |
| MES-SA | Etoposide | | | | | 4.7 | 1.5 |
| 05-1F11 | (1.9) | 2.2 | (–10)[d] | (–9.1)[4] | (1)[d] | 0.9 (–5.5 ± 1.8) | 1.5 |
| 1-4G11 | (3.4) | 5.0 | (–33)[d] | (–20)[4] | (1)[d] | 1.7 (–2.8 ± 0.7) | 1.2 |

[a]Fold sensitivity given for adherent cells only, based on $D_{37}$ values. [b]Relative mRNA expression levels as determined by densitometric scanning of autoradiographs and equalized according to the GAPDH loading control. [c]Relative protein expression as determined by densitometric scanning of Western blots. [b,c]Based on data from at least three independent experiments, fold changes in expression of mRNA and protein levels shown in parentheses (+ increase, – decrease), only shown if greater than 2.5, ± standard errors. [d]Data from Jaffrezou et al (1994).

© Cancer Research Campaign 1997 

**Figure 1** RNAase protection assay to quantify topo IIα and -β mRNA (**A**) and MRP mRNA (**B**) levels in human cancer cell lines and their resistant counterparts. The samples are arranged in pairs with the parental line on the left and its resistant derivative on the right, as indicated in each case above the lanes. The positions of the protected fragments are indicated on the left. The sizes of the molecular weight standards (in base pairs) are shown on the right. Densitometric scanning of autoradiograph was performed when each signal was within the linear range for radiographic film

β isoform. In contrast, the CEM/MX1 subline showed no alteration in topo IIα gene expression but a 10- to 20-fold down-regulation of the topo IIβ mRNAs (Figure 1A). None of the

**Figure 2** Western blot analysis to quantify the level of expression of topo IIα (**A**) and β (**B**) proteins in nuclear protein extracts. Samples are shown arranged in pairs with the parental line on the left in each case and its resistant derivative on the right, as indicated above the lanes. The position of the 200 kDa molecular weight standard is shown on the left aforementioned drug-resistant derivatives showed an altered level of MRP mRNA expression. In contrast, while the U1285-dox800 resistant cell line showed no significant change in the level of topo IIα or -β mRNA expression, it did exhibit a 30-fold overexpression of the MRP gene (Figure 1B). The MES-SA cell sublines, derived by single-step exposure to low doses of etoposide, have been reported previously to show down-regulation of both topo IIα and -β mRNAs, with no alterations in MDR1 or MRP gene expression (Jaffrezou et al, 1994). Thus, the drug-resistant cell lines studied showed no consistent pattern of altered topo II gene expression, although down-regulation of the mRNA for one or the other (or both) topo II isoforms was a general feature of this cell line panel (Table 1).

**Figure 3** Clonogenic survival curves for parental cell lines and their corresponding resistant counterparts following exposure to increasing concentrations of ICRF-159. (**A**) SuSa (○), MCF-7 (□) and NCI-460 (△) parental cell lines and SuSa/VC2 (●), MCF-7 ADr[R] (■) and 460pV8 (▲) resistant sublines. (**B**) MES-SA parental cell line (▲) and the resistant sublines, 05-1F11 (▽) and 1-4G11 (△). Points represent the mean of three independent experiments

 © Cancer Research Campaign 1997

**Figure 4** Growth inhibition assay for parental U-1285 cells and CEM cells (**A**) and (**C**) respectively) and the drug-resistant U-1285/dox800 and CEM/MX1 (**B** and **D** respectively) cells during exposure to increasing doses of ICRF-159. In each case, the symbols represent the following doses of ICRF-159: ○, no drug; ●, 4 μg $ml^{-1}$; ▲, 8 μg $ml^{-1}$ and □, 16 μg $ml^{-1}$

The differing patterns of mRNA expression in the resistant variants was reflected in levels of topo II protein expression, as determined by Western blotting of nuclear protein extracts (Figure 2; Table 1). A similar analysis using whole-cell extracts gave equivalent results (data not shown). However, the degree of protein down-regulation in each resistant variant was generally less marked than was the degree of mRNA down-regulation. Indeed, only in the case of the MES-SA cell-resistant derivatives was the degree of topo II protein down-regulation greater than 2.5-fold (Table 1).

### Measurement of sensitivity to ICRF-159

In order to address whether the panel of drug-resistant cell lines showed an altered response to a non-cleavable complex-forming topo II-targeting agent, the parental and resistant variants were tested for their relative sensitivity to ICRF-159. The data in Figures 3 and 4 show that none of the resistant cell lines studied was cross-resistant to ICRF-159. Indeed, the MES-SA 05-F11 and 1-4G11 cell lines showed collateral sensitivity to ICRF-159. These

© Cancer Research Campaign 1997

MES-SA-derived cell lines exhibit down-regulation of both topo II isoforms at the mRNA level (Jaffrezou et al, 1994), whereas at the protein level only the α isoform appears to be significantly down-regulated (Figure 2; Table 1).

### Relationship between topo II expression and sensitivity to topo II-targeting drugs

In agreement with many reports, our results show that a decrease in the level of the cellular target (topo II) is associated with relative resistance to cleavable complex-forming drugs (reviewed in Beck et al, 1993). This is because the cytotoxicity of these agents is as a result of their ability to subvert topo II from its normal physiological role, in which DNA cleavage occurs only transiently, to one in which potentially cytotoxic double-stranded DNA breaks persist in the DNA. Conversely, down-regulation of the target enzyme might be expected to confer hypersensitivity to non-cleavable complex-forming topo II-targeting drugs, such as ICRF-159, which act as direct inhibitors of the catalytic activity of the enzyme. Such a relationship between a 'catalytic inhibitor' and its target enzyme is well established for drugs that target dihydrofolate reductase (reviewed in Fairchild et al, 1990) and thymidylate synthase (Freemantle et al, 1995). Our study has shown that cell lines selected for resistance to cleavable complex-forming topo II inhibitors are not cross-resistant to ICRF-159. This is the case for a series of cell lines of different tissue origin, some of which exhibit a multidrug-resistant phenotype. This would indicate that overexpression of P-glycoprotein, MRP or certain classes of GSTs does not by itself confer resistance to ICRF-159.

Collateral sensitivity to ICRF-159 was seen only in the MES-SA 05-1F11 and 1-4G11 cell lines, despite the finding that some of the other representatives of this cell line panel showed a modest level of down-regulation of topo II protein. However, the MES-SA cell lines did show the greatest degree of topo IIα protein down-regulation. Despite this, there was no correlation between the extent of topo IIα down-regulation and the degree of ICRF-159 sensitivity in the MES-SA cell lines. Thus, clone 05-1F11 displayed a lower level of topo IIα protein than did clone 1-4G11, but both cell lines had similar ICRF-159 sensitivity. Nevertheless our results are consistent with the notion that the likely target for ICRF-159 is the topo IIα protein in human cells and that down-regulation of the α isozyme alone can confer sensitivity to ICRF-159, as the dramatic down-regulation of the β isoform in the CEM/MX1 subline did not appear to influence the degree of ICRF-159 sensitivity. Clearly, further work will be required to confirm this suggestion. If this proves to be correct, our results suggest that sensitivity to ICRF-159 may only occur when the nuclear content of topo IIα falls below a critical threshold level as members of the cell line panel other than 05-1F11 and 1-4G11 exhibited a small down-regulation of topo IIα. The level that is set for this threshold might be dependent upon the requirement in each cell line for a particular level of topo IIα activity during chromosome segregation at mitosis.

Cells of testicular germ cell origin are very sensitive, in vivo and in vitro, to a wide range of drugs, including *cis*-platinum and bleomycin. However, the testicular teratoma cell line, SuSa, was more resistant to ICRF-159 than any of the other parental cell lines studied. The high basal topo II activity in the parental SuSa cell line (Fry et al, 1991) might be responsible for the intrinsic resistance of this line to ICRF-159 compared with other epithelial cell lines.

In the clinical situation, where resistance to cleavable complex-forming topo II-targeting drugs is encountered all too frequently (although whether this is conferred by changes in topo II is not known), it would be interesting to analyse whether patients still respond to treatment with topo II-targeting drugs, such as ICRF-159, which act via a distinct mechanism. Bis(2,6-dioxopiperazine) derivatives have previously been shown to have some anti-tumour activity in leukaemia and sarcomas. Marrow suppression was the dose-limiting toxicity in Phase II studies, and use of the drug was generally discontinued (Tsukagoshi, 1994). Re-evaluation of this drug (or an analogous agent) may be indicated, in combination with marrow support provided by colony-stimulating factors.

### ACKNOWLEDGEMENTS

We thank Dr S Cole for the MRP RNAase protection probe and Drs K Cowan, G Harker, B Hill, S Houlbrook and B Sikic for providing cell lines. Dr A Creighton generously provided ICRF-159 We also thank the Imperial Cancer Research Fund for financial support.

### REFERENCES

Ausubel FM (1994) *Current Protocols in Molecular Biology*. John Wiley and Sons: New York

Batist G, Tulpule A, Sinha BK, Katki AG, Myers CE and Cowan K (1986) Overexpression of a novel anionic glutathione transferase in multidrug-resistant human breast cancer cells. *J Biol Chem* **261**: 15544–15549

Beck WT, Danks MK, Wolverton JS, Kim R and Chen M (1993) Drug resistance associated with altered DNA topoisomerase II. *Advan Enzyme Regul* **33**: 113–127

Bradley G and Ling V (1994) P-glycoprotein, multidrug resistance and tumour progression. *Cancer Metastasis Rev* **13**: 223–233

Chen M and Beck W (1995) Differences in inhibition of chromosome separation and G2 arrest by DNA topoisomerase II inhibitors merbarone and VM-26. *Cancer Res* **55**: 1509–1516

Chomczynski P and Sacchi N (1987) Single-step method of RNA isolation by acid guanidinium thiocyanate–phenol–chloroform extraction. *Anal Biochem* **162**: 156–159

Cole SPC, Bhardwaj G, Gerlach JH, Mackie JE, Grant CE, Almquist KC, Stewart AJ, Kurz EU, Duncan AMV and Deeley RG (1992) Overexpression of a transporter gene in a multidrug resistant human lung cancer cell line. *Science* **258**: 1650–1654

Danks MK, Warmouth MR, Friche E, Granzen B, Bugg BY, Harker G, Zwelling LA, Futscher BW, Suttle DP and Beck WT (1993) Single-strand conformational polymorphism analysis of the Mr 170,000 isozyme of DNA topoisomerase II in human tumour cells. *Cancer Res* **53**: 1373–1379

Davies SL, Jenkins JR and Hickson ID (1993) Human cells express two differentially spliced forms of topoisomerase IIβ messenger RNA. *Nucl Acids Res* **21**: 3719–3723

Davies SM, Robson CN, Davies SL and Hickson ID (1988) Nuclear topoisomerase II levels correlate with the sensitivity of mammalian cells to intercalating agents and epipodophyllotoxins. *J Biol Chem* **263**: 17724–17729

Fairchild CR, Goldsmith ME and Cowan KH (1990) *Molecular Biology of Antineoplastic Drug Resistance* Cossman J (ed.), pp. 113–141. Elsevier: New York

Freemantle SJ, Jackman AL and Kelland LR (1995) Molecular characterisation of two cell lines selected for resistance to the folate-based thymidylate synthase inhibitor, ZD1694. *Br J Cancer* **71**: 925–930

Froelich-Ammon S and Osheroff N (1995) Topoisomerase poisons: harnessing the dark side of enzyme mechanism. *J Biol Chem* **270**: 21429–21432

Fry AM, Chresta CM, Davies SM, Walker MC, Harris AL, Hartley JA, Masters JRW and Hickson ID (1991) Relationship between topoisomerase II level and chemosensitivity in human tumour cell lines. *Cancer Res* **51**: 6592–6595

Glisson B, Gupta R, Smallwood-Kentro S and Ross W (1986) Characterization of acquired epipodophyllotoxin resistance in a chinese hamster ovary cell line: loss of drug-stimulated DNA cleavage activity. *Cancer Res* **46**: 1934–1938

© Cancer Research Campaign 1997

Hoskins L, Whelan R, Shellard S, Davies S, Hickson I, Danks M and Hill B (1994) Multiple mechanisms of resistance in a series of human testicular teratoma cell lines selected for increasing resistance to etoposide. *Int J Cancer* **57**: 259–267

Houlbrook S, Addison C, Davies S, Carmichael J, Stratford I, Harris A and Hickson I (1995) Relationship between expression of topoisomerase II isoforms and intrinsic sensitivity to topoisomerase II inhibitors in breast cancer cell lines. *Br J Cancer* **72**: 1454–1461

Jaffrezou J, Chen G, Duran G, Kuhl J-S and Sikic B (1994) Mutation rates and mechanisms of resistance to etoposide determined from fluctuation analysis. *J Natl Cancer Inst* **86**: 1152–1158

Jenkins JR, Ayton P, Jones T, Davies SL, Simmons DL, Harris AL, Sheer D and Hickson ID (1992) Isolation of cDNA clones encoding the β isozyme of human DNA topoisomerase II and localisation of the gene to chromosome 3p24. *Nucl Acids Res* **20**: 5587–5592

Laemmli UK (1970) Cleavage of structural proteins during the assembly of the head of bacteriophage T4. *Nature* **227**: 680–685

Liu LF, Rowe TC, Yang L, Tewey KM and Chen GL (1983) Cleavage of DNA by mammalian DNA topoisomerase II. *J Biol Chem* **258**: 15365–15370

Moscow JA, Fairchild CR, Madden MJ, Ransom DT, Wieand H, O'Brien EE, Poplack DG, Cossman J, Myers CE and Cowan KH (1989) Expression of anionic glutathione S-transferase and P-glycoprotein genes in human tissues and tumors. *Cancer Res* **49**: 1422–1428

Pommier Y (1993) DNA topoisomerase I and II in cancer chemotherapy: update and perspectives. *Cancer Chemother Pharmacol* **32**: 103–108

Roca J, Ishida R, Berger JM, Andoh T and Wang JC (1994) Antitumour bisdioxopiperazines inhibit yeast DNA topoisomerase II by trapping the enzyme in the form of a closed protein clamp. *Proc Natl Acad Sci USA* **91**: 1781–1785

Tanabe K, Ikegami Y, Ishida R and Andoh T (1991) Inhibition of topoisomerase II by antitumour agents bis(2,6-dioxopiperazine) derivatives. *Cancer Res* **51**: 4903–4908

Tsugoshi S (1994) A novel antitumor agent, sobuzoxane (MST-16). *Jpn J Cancer Chemother* **21**: 1089–1097

© Cancer Research Campaign 1997